# Novel Film-Forming Spray: Advancing Shelf Life Extension and Post-Harvest Loss Reduction in Eggs

**DOI:** 10.3390/polym17152142

**Published:** 2025-08-05

**Authors:** Nagesh Sonale, Rokade J. Jaydip, Akhilesh Kumar, Monika Madheswaran, Rohit Kumar, Prasad Wadajkar, Ashok Kumar Tiwari

**Affiliations:** 1 ICAR—Central Avian Research Institute, Izatnagar, Bareilly 243122, Uttar Pradesh, India; dr.nssonale@gmail.com (N.S.); prasadwadajkar@gmail.com (P.W.); ashok.tiwari1@icar.gov.in (A.K.T.); 2 Division of Medicine, ICAR—Indian Veterinary Research Institute, Izatnagar, Bareilly 243122, Uttar Pradesh, India; rajputrohit883@gmail.com; 3 ICAR—Indian Agriculture Research Institute, Hazaribag 825405, Jharkhand, India; monika.m@icar.gov.in

**Keywords:** egg preservation, phytoconstituents, antimicrobial properties, sustainability, poultry industry

## Abstract

This study explores the development of a topical film-forming spray infused with phytobiotic herbs to extend egg shelf life and maintain its quality. Unlike traditional surface treatments, film-forming sprays provide uniform drug distribution, better bioavailability, effective CO_2_ retention by sealing pores, and antibacterial effects. The spray includes a polymer to encapsulate phytoconstituents and form the film. The resulting film is highly water-resistant, glossy, transparent, and dries within two minutes. SEM analysis showed a fine, uniform morphology, while zeta analysis revealed a negative potential of −0.342 mV and conductivity of 0.390 mS/cm, indicating stable dispersion. The spray’s effectiveness was tested on 640 chicken eggs stored at varying temperatures. Eggs treated and kept at 2–8 °C showed the best results, with smaller air cells, higher specific gravity, and superior quality indicators such as pH, albumen weight, albumen height and index, Haugh unit, yolk weight, and yolk index. Additionally, the spray significantly reduced microbial load, including total plate count and *E. coli*. Eggs stored at 28 °C remained safe for 24–30 days, while those at 2–8 °C lasted over 42 days. This innovative film-forming spray offers a promising approach for preserving internal and external egg quality during storage.

## 1. Introduction

India’s agricultural and industrial revolutions have significantly contributed to sectoral development and economic resilience [1]. Notably, the Silver Revolution spurred rapid advancements in poultry and egg production, positioning India as the world’s third-largest egg producer, with an annual output of 138.38 billion eggs, and a major broiler producer [2]. However, despite such growth, the Indian egg industry faces persistent challenges in meeting domestic nutritional demands, largely due to post-harvest losses (PHLs) occurring across the supply chain, from production and transportation to marketing and storage [3].

This issue is not unique to India; globally, post-harvest losses in eggs and perishable animal products remain a major concern, especially in low- and middle-income countries where infrastructure, cold chain systems, and handling practices are often inadequate [4]. According to the Food and Agriculture Organization (FAO), up to 20–25% of animal-based foods are lost or wasted globally due to poor post-harvest practices [5]. In the context of eggs, losses can include breakage, microbial contamination, and the degradation of quality due to exposure to moisture, temperature fluctuations, and physical mishandling. In India, the lack of appropriate handling systems, inadequate storage conditions, and weak transport infrastructure further exacerbate these losses. These not only affect food security and economic returns for farmers but also contribute to environmental burdens through increased waste. Therefore, innovative, scalable interventions such as protective sprays or edible coatings hold promise not only for India but also for other egg-producing nations facing similar logistical and post-harvest challenges [6]. Addressing PHL through affordable and accessible technologies is critical to improving global food system sustainability, reducing waste, and enhancing the availability of high-quality protein sources worldwide [7].

Eggs are a rich source of high-quality proteins, vitamins, antioxidants, carotenoids, and phospholipids, making them essential to the human diet. Chicken eggs are especially valued for their nutritional profile and widespread consumption [8]. However, due to aging, egg quality begins to decline immediately after laying, which affects internal components. Factors such as hen age, diet, disease, environmental conditions, handling, and storage play critical roles in this process. Prolonged storage, particularly at ambient temperatures, leads to CO_2_ loss through the shell, causing deteriorations in albumen, yolk, weight, and pH [9].

Coating techniques are crucial in prolonging shelf life and maintaining food quality by regulating mass transfer, moisture diffusion, and gas permeability (O_2_ and CO_2_) while preserving mechanical and rheological properties [10]. Traditional egg preservation techniques for extending the shelf life, such as oil coating and water glass immersion, help form a protective barrier against degradation [11]. However, these methods present practical limitations—oil coating requires meticulous application due to its messy nature. At the same time, water glass immersion demands precise preparation and may negatively impact egg quality, making both methods less viable for large-scale commercial use. Edible coatings from various materials such as biopolymers derived from natural sources including polysaccharides, lipids (such as waxes and glycerides), and proteins (such as gelatin and collagen) have gained attention for their potential benefits [12]. The effectiveness of an egg-coating material depends on factors such as temperature and storage duration [13,14]. In the poultry industry, coating materials are classified as lipid-, protein-, or polysaccharide-based including starch carboxymethyl cellulose (CMC), carboxymethyl chitosan, carboxymethyl bacterial cellulose, sericin, keratin, fibroin, and pectin [15]. Plasticizers such as glycerol, polyethene glycol (PEG), and sorbitol are often added to enhance coating flexibility and durability. Additionally, composite coatings have been developed to improve gas exchange, adhesion, and moisture vapor permeability, further enhancing coating effectiveness [16].

This study introduces an innovative film-forming spray to extend chicken eggs’ shelf life under different storage conditions. The formulation addresses storage-related losses through a multifunctional composition with a protective polymer coating [17], a synergistic blend of phytobiotics, and an emulsifier for optimal integration. This coating enhances egg freshness, quality, and longevity under various temperatures. By providing an effective and sustainable method for extending egg shelf life, this spray formulation has the potential to enhance storage practices, reduce post-harvest losses, and improve consumer satisfaction.

## 2. Materials and Methods

### 2.1. Materials and Samples

The phytobiotic used in this study, classified as a monoterpene aldehyde, was procured from Elixir Extracts Pvt. Ltd., Kerala, India. (Note: The specific identity of the phytobiotic is not being disclosed due to intellectual property protection; patent application no. 202311066448.) This bioactive compound is known to exert potent antimicrobial effects by disrupting the permeability of microbial cell membranes, precipitating cytoplasmic contents, and interfering with essential intracellular processes. Other reagents used in the formulation included polyvinyl alcohol (PVA) (molecular weight 30,000–70,000), polyethylene glycol (PEG-400), and ethanol, all sourced from Sigma-Aldrich, Merck, Germany.

### 2.2. Preparation of Film-Forming Spray

To prepare the film-forming spray, we dissolved an amount (2–7%) of phytobiotic in ethanol. We dissolved PVA (2–7% (*w*/*v*)) in distilled water (23–61%) at 450 rpm, 45 °C for 30 min, followed by increasing the stirring speed to 550 rpm and lowering the temperature from 45 °C to 28 °C for another 10 min. During this, ethanol (35–65% *v*/*v*) was added in a dropwise manner, followed by the addition of phytobiotic solution (1–3% (*w*/*v* or *v*/*v*)) and PEG (0.5–2.0% *v*/*v*) under continued stirring conditions. The resultant preparation was sonicated in ice at a frequency of 10 Hz for three 10 s cycles with 30 s breaks and stored at 4 °C until further use.

### 2.3. Experimental Design

In this experiment, 640 fresh eggs from the CARI Priya breed were collected from the layer farm at the Indian Council of Agricultural Research (ICAR)—Central Avian Research Institute (CARI), Izatnagar, Bareilly (UP), India. The eggs were divided into four treatment groups, each containing 160 eggs. Each treatment group was divided into four replicates, with 40 eggs per replicate. Details of the treatment groups are provided in Supplementary Table S1. To determine optimal storage conditions, including temperature and duration, external and internal quality parameters were assessed at specific intervals: 0, 6, 12, 18, 24, 30, 36, and 42 days. This study also aimed to identify post-harvest losses associated with egg storage.

### 2.4. Evaluation of Physical Appearance Parameters of Film-Forming Spray

#### 2.4.1. Water Washability Test

For the evaluation of the film’s water washability and wetting properties, a few drops of the spray solution were applied to a clean glass slide and allowed to dry completely at room temperature (33–35 °C) for 12–15 h. Once dried, the slide was placed under slow-running water for one minute, and its washability was assessed using an ordinal scale: easily washed, moderately washed, or poorly washed [18].

#### 2.4.2. Cosmetic Appearance Analysis

The cosmetic appearance of the film was assessed by applying the spray solution to a clean, dry glass slide and allowing it to dry. The dried film was then visually examined and categorized based on its shine and transparency as low (shiny and transparent), medium (shiny and translucent), or high (dull and opaque) [18].

#### 2.4.3. Integrity Evaluation

To assess film integrity, the spray solution was applied to a clean slide and left at room temperature (33–35 °C) for 12 h. The dried film was then visually inspected and classified as cracked, flaking, or intact, providing insight into its durability and longevity [19].

#### 2.4.4. Viscosity

The viscosity of the spray solution was evaluated by forming a thin film on a clean surface and allowing it to dry completely. Once dried, a piece of cotton wool was gently pressed onto the film, and the number of fibers adhering to the surface was observed. Viscosity was determined based on fiber adhesion [19,20]:

- High viscosity: Many fibers adhered, indicating strong adhesiveness;
- Moderate viscosity: A moderate number of thin fibers attached;
- Low viscosity: Few or no fibers adhered, indicating low adhesiveness.

### 2.5. Qualitative Evaluation of Film-Forming Spray

#### 2.5.1. pH Measurement

The pH of the film-forming spray solution was determined by taking 2 mL of the solution in a 20 mL glass beaker and measuring it using a digital pH meter [21].

#### 2.5.2. Evaporation Time

This parameter assesses formulation consistency and drying efficiency. The prepared solution was applied to plain white paper and left to dry to determine the evaporation time. The time required for complete drying was recorded [18].

#### 2.5.3. Drying Time Assessment

Approximately 10 µL/cm^2^ of the spray solution was applied to a clean glass slide without spreading to measure the drying time. The solution was left to dry naturally, and the time was recorded once no visible liquid remained upon physical examination [19,20].

#### 2.5.4. Thickness Measurement

Film thickness is a crucial parameter for evaluating in vivo behavior. A few drops of the spray solution were applied to a clean glass slide and allowed to dry at room temperature (33–35 °C) for 6–8 h. Once dried, the film was carefully removed and measured using a digital thickness gauge, with thickness recorded in millimetres (mm) [19,20].

#### 2.5.5. Volume per Stroke

The volume of solution delivered per stroke was measured by spraying the solution 10 times into a graduated tube. The total collected volume was recorded, and the average volume per stroke was calculated [22].

### 2.6. Physicochemical Characterization of Film-Forming Spray

Advanced analytical techniques were utilized, including a zeta sizer for measuring vesicle size, Fourier Transform Infrared Spectroscopy (FTIR) (Thermo Scientific Nicolet 912A0715 iS5, Vernon Hills, IL, USA) for the detailed characterization of the chemical functional groups, and Scanning Electron Microscopy (SEM) (JSM-IT200, Jeol, Tokyo, Japan) for morphological analysis.

#### 2.6.1. Measurement of Zeta Size and Potential

The film-forming spray’s hydrodynamic size and zeta potential were measured using a zeta sizer to assess its stability and surface charge (Anton Parr Litesizer 500, Graz, Austria). The instrument was zeroed with 500 µL of 30% ethanol.

#### 2.6.2. FTIR Analysis

FTIR analysis (Thermo Scientific Nicolet 912A0715 iS5, Vernon Hills, IL, USA) was performed for all formulations by scanning the samples across a 4000–400 cm^−1^ wavelength range and was used to identify the functional groups and confirm the presence of encapsulated compounds in the spray.

#### 2.6.3. Scanning Electron Microscope Analysis

SEM (JSM-IT200, Jeol, Japan) was used to analyze the surface morphology of the film-forming spray. The detailed imaging provided insights into the film’s structural integrity, uniformity, and potential barrier properties.

### 2.7. Evaluation of Effect of Film-Forming Spray on External Egg Quality Parameter

The following procedure was followed to evaluate the effect of a single spray on external egg quality parameters, including egg weight loss %, air cell diameter, eggshell thickness, and specific gravity.

#### 2.7.1. Egg Weight Loss (%)

The egg weight loss was measured weekly using the initial egg weight recorded on Day 0, following the equation provided by Paula et al. [23]. The effects of the film-forming spray on the percentage of egg weight loss were evaluated under different storage durations and temperatures.

$$Weight\;loss\;\left(\%\right)=\frac{Final\;weight\;\left(g\right)-Initial\;weight\;\left(g\right)\;}{Initial\;weight\;\left(g\right)}\times 100$$

#### 2.7.2. Air Cell Diameter

The air cell diameter of an egg can be accurately measured by positioning the egg under a candling lamp and using a Vernier caliper to measure the diameter of the air cell. According to Stadelman (1995), the following formula is commonly applied [24,25]:

$$Air\;cell\;diameter=\frac{Width\;}{Diameter\;of\;the\;air\;cell\;visible\;under\;candling}$$

#### 2.7.3. Specific Gravity

Specific gravity was calculated according to the equation provided by Çelik et al. (2021) [26]. This involved measuring the egg’s weight in both air and distilled water using an analytical balance with a sensitivity of 0.01 g [26,27].

$$\mathrm{Specific}\;\mathrm{gravity}=\frac{Weight\;in\;Air\;\left(g\right)}{Weight\;in\;Air\;\left(g\right)-Weight\;in\;water\;\left(g\right)}$$

#### 2.7.4. Shape Index

The egg’s maximum length and breadth were precisely measured using a Vernier caliper, and the shape index was subsequently calculated following the following formula [28,29]:

$$\mathrm{Shape}\;\mathrm{Index}\;=\frac{Max.\;breadth\;of\;egg\;\left(cm\right)}{Max.\;length\;of\;egg\;\left(cm\right)}\times 100$$

#### 2.7.5. Eggshell Thickness

Eggshell thickness is measured using specialized equipment, such as an eggshell thickness gauge. For the calculation of eggshell thickness, the average of three different edges ( viz broad, middle, and narrow) was measured [30].

### 2.8. Evaluation of Effect of Film-Forming Spray on Internal Egg Quality Parameter

Various internal egg quality parameters were analyzed at different ages, including albumen, yolk, whole egg pH, Haugh unit, albumen height, yolk weight (%), index, and color [31].

#### 2.8.1. pH of Albumen, Yolk, and Whole Egg

The yolk of sprayed eggs was separated from the albumen, and both were distributed into three replicates of glass beakers. The pH determination of the albumen, whole egg, and yolk was performed using a digital pH meter (Electronic Instrument Ltd., Chennai, India). About 2.0 g of the sample was homogenized in 20.0 mL of de-ionized water in a beaker. The pH meter was first calibrated using pH 4.01 and 9.20 buffer solution. The electrode was then rinsed with de-ionized water and dipped into the homogenate, allowing sufficient time for stabilization before taking the reading [32].

#### 2.8.2. Haugh Unit, Albumen Height and Weight

Haugh unit is the most widely used parameter for albumen quality. The height of the albumen (mm) and egg weight (g) of film-forming sprayed eggs were used for the calculation of the Haugh unit using the following formula described by Haugh, 1937 [33,34]. After measuring the height and width of the thick albumen, the albumen was separated carefully from the yolk using a blunt knife, and its weight was measured using a weighing balance with 0.5 mg accuracy [35].

Haugh Unit =100logH +7.57−1.7W^0.37

where H = albumen height (mm), W = weight of egg (g).

#### 2.8.3. Yolk’s Weight (%), Index, and Color

After separating the yolk and albumen, the spherical nature of the yolk can be expressed as a yolk index by keeping the yolk intact. An AMES spherometer was used to measure the height of the yolk and a Vernier caliper was used to measure its width. The following formula was used to calculate the yolk index is described below. Further yolk color was measured by using a yolk color fan [36].

$$\mathrm{Yolk}\;\mathrm{Index}\;=\frac{Yolk\;Height\;\left(mm\right)}{Yolk\;Width\;\left(mm\right)}\times 100$$

### 2.9. Evaluation of Effect of Film-Forming Spray on Microbial Quality

For total microbial count (TPC) analysis, the egg content was diluted in a 1:9 ratio with buffered peptone water (BPW) and homogenized using a vortex mixer for two minutes. The homogenized sample was then plated on Plate Count Agar (PCA) and incubated at 34–36 °C for 48 h [37]. After incubation, the bacterial colonies were counted and multiplied by the reciprocal of the dilution factor, with results expressed as colony-forming units per millilitre (cfu/mL). Similarly, to assess changes in *E. coli* populations, we performed spread-plating by inoculating 0.1 mL of the appropriate dilutions onto Eosin Methylene Blue (EMB) agar [38]. The egg content and surface solutions were serially diluted in PBS before plating. Following 48 h incubation at 37 °C, *E. coli* colonies were enumerated to evaluate population dynamics. The film-forming spray was also evaluated on two field strains of *Salmonella typhimurium* of poultry origin (2959 and 22NSC003) provided by the Salmonella Laboratory of ICAR-Indian Veterinary Research Institute, Izatnagar. The revived stains were spread evenly on a nutrient agar plate along with the spotting of the film-forming spray in a concentration of 0.25 mg, 0.50 mg, 0.75 mg and 1.0 mg, respectively, and incubated at 37 °C for 24 h.

### 2.10. Data Analysis

All data obtained from this study were subjected to a one-way and two-way ANOVA using statistical software, using IBM, statistical software package SPSS, version 26.0. The means were compared for significance using the Duncan range test. *p* ≤ 0.05 was considered statistically significant.

## 3. Results

### 3.1. Formulation and Optimization of Film-Forming Spray

A film-forming spray was formulated using four ingredients in six distinct compositions (F1–F6) (Supplementary Figure S1), each evaluated for key characteristics. The assessments covered physical appearance, including water washability, cosmetic appeal, integrity, and viscosity (Table 1), followed by qualitative parameters such as evaporative time, drying time, pH, volume per spray, and film thickness.

Washability tests classified F1 and F2 as easily washable (Grade 1), F3 and F4 as moderately washable (Grade 2), and F5 and F6 as highly water-resistant (Grade 3). The cosmetic evaluation confirmed all formulations exhibited shininess and transparency (Grade 2). Film integrity varied, with F1 and F2 showing moderate integrity (flaking, Grade 2), while F3–F6 maintained intact films (Grade 3). Viscosity ranged from water-like (F1–F3, Grade 1) to glycerol-like (F4, F5, Grade 2) and syrup-like (F6, Grade 3).

All formulations produced transparent to translucent films, with F5 emerging as the most acceptable. The pH ranged from 5.57 to 5.87, ensuring compatibility with the intended application. Evaporation times varied from 30 to 55 min, while drying times ranged from 1.31 min (F1) to 3.37 min (F2), affecting application practicality. Each spray delivered a uniform 0.1 mL dose, forming films with thicknesses between 0.03 mm and 0.12 mm (Table 2).

Based on superior physical and qualitative attributes, F4 and F5 were selected for further characterization, including zeta size, potential, FTIR, and SEM analysis.

### 3.2. Physicochemical Characterization

#### 3.2.1. Measurement of Zeta Size and Potential

**Figure 1:**
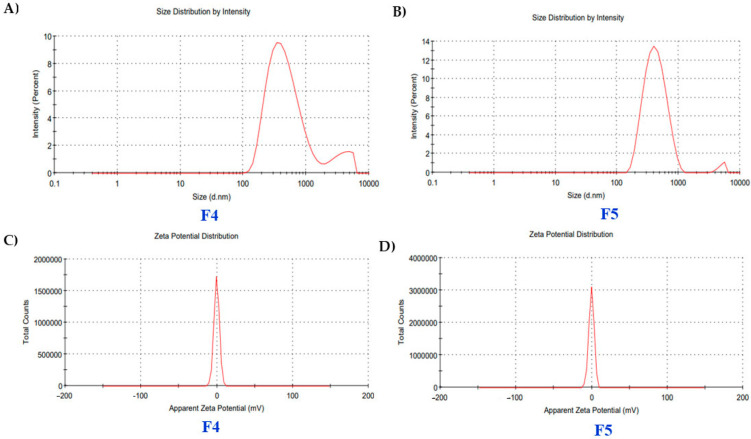
(**A**,**B**) Zeta size and (**C**,**D**) zeta potential analysis of film-forming spray (F4 and F5).

Zeta size analysis demonstrates consistent nanoscale distributions. F4 exhibits a Z-average of 43.5 nm with a low polydispersity index (PDI) of 0.234, indicating a narrow size range and a dominant peak at 43.5 nm with 99.9% intensity. F5 has a slightly smaller Z-average of 35.1 nm, a broader PDI of 0.269, and a primary peak at 42.4 nm with 97.4% intensity (Figure 1A,B). F4 has a zeta potential of −0.31 mV and conductivity of 0.402 mS/cm. In comparison, F5 exhibits a slightly more negative zeta potential of −0.342 mV with a conductivity of 0.390 mS/cm, indicating stable dispersion properties (Figure 1C,D).

#### 3.2.2. FTIR Analysis

**Figure 2:**
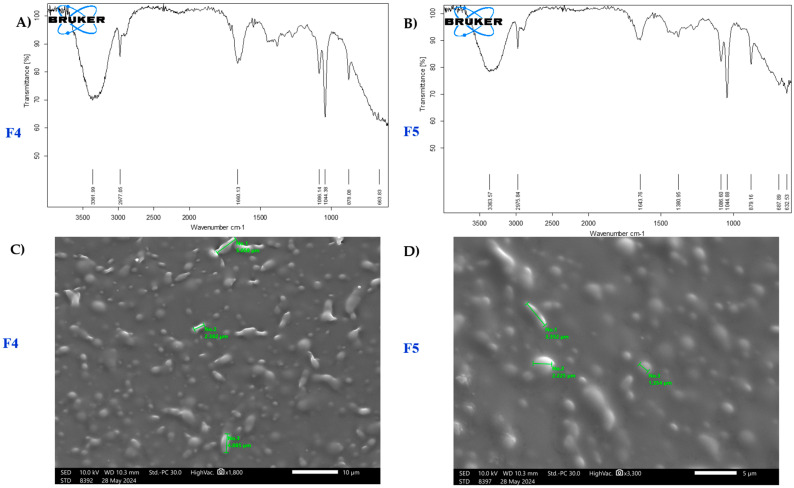
(**A**,**B**) FTIR analysis of film-forming spray F4 and F5 and (**C**,**D**) morphology of film-forming spray F4 and F5 under SEM.

The FTIR spectra of formulations F4 and F5 reveal key functional groups based on their transmittance peaks (Figure 2A,B). The broad absorption peak around 3300 cm^−1^ in both spectra indicates O-H stretching, likely from hydroxyl or alcohol groups, suggesting similar chemical compositions. Peaks near 2900 cm^−1^ correspond to C-H stretching, characteristic of aliphatic chains, further confirming shared structural components.

#### 3.2.3. SEM Analysis

SEM analysis (Figure 2C,D) highlights particle morphology and distribution differences. F4 features irregularly shaped particles with an average size of 1.86 µm and a broader distribution (1.83–2.43 µm). In contrast, F5 presents a more homogeneous morphology with primary particle sizes of 1.13 µm and 1.54 µm, suggesting a finer and more uniform structure. The denser, compact arrangement in F5 indicates enhanced structural integrity, making it better suited for applications requiring consistent particle morphology.

### 3.3. Effect of Phyto-Biotic Spray on External Parameters of Egg Quality During Storage

#### 3.3.1. Egg Weight Loss (%)

The effect of the film-forming spray on egg weight loss was evaluated under different storage durations and temperatures (Table 3). The results showed significant (*p* < 0.001) differences among treatment groups. At Day 42, weight loss was highest in T1 (15.58%) and lowest in T4 (4.52%), with T2 (6.99%) and T3 (8.87%) in between. Higher temperatures increased weight loss, while the spray helped retain egg quality. T4 had the least weight loss, confirming the positive impact of both lower temperatures and the film-forming spray.

#### 3.3.2. Air Cell Diameter and Specific Gravity

Statistical analysis (*p* < 0.05) confirmed that spray and refrigeration slowed air cell expansion (Table 4). At 28 °C without spray (T1), the diameter increased from 16.48 mm to 33.68 mm, while sprayed eggs at 2–8 °C (T4) showed minimal expansion (25.44 mm), highlighting their combined efficacy in freshness retention. Eggs at 28 °C without spray (T1) exhibited a significant (*p* < 0.001) decline in specific gravity (1.092 to 1.04), indicating rapid moisture loss. In contrast, sprayed eggs at 2–8 °C (T4) maintained better integrity (1.094 to 1.066), proving the synergistic effect of refrigeration and spray (Table 4).

#### 3.3.3. Shape Index

Supplementary Table S2 shows the influence of the film-forming spray and storage temperature on the shape index of eggs over 42 days, with comparisons between sprayed and non-sprayed eggs under varying storage conditions. The results found that the film-forming spray and different storage temperatures had no significant effect on the shape index of eggs over 42 days (*p* > 0.05). Across all treatments, the shape index remained relatively stable, with only minimal fluctuations, indicating that the film-forming spray and temperature variations did not impact the egg shape. These findings suggest that the treatments effectively preserve egg quality without altering structural integrity.

#### 3.3.4. Effect of Film-Forming Spray on Egg Shell Thickness

The effect of the film-forming spray on eggshell thickness was assessed under two storage temperatures (28 °C and 2–8 °C) over various durations with and without spray, as represented in Supplementary Table S3. The investigation into the effects of the film-forming spray on eggshell thickness under varying storage temperatures (28 °C and 2–8 °C) revealed no significant (*p* > 0.05) differences between the sprayed and non-sprayed groups across the different durations.

### 3.4. Effect of Film-Forming Spray on Internal Parameters of Egg Quality During Storage

#### 3.4.1. Albumen, Yolk, and Whole Egg pH

At 28 °C without spray (T1), albumen pH rose from 7.86 to 9.82, accelerating alkalinization (*p* < 0.05). Refrigeration (T2) slowed this increase, while the film-forming spray further stabilized pH, especially in T4 (7.88 to 8.00), underscoring its protective potential (Table 4). Yolk pH increased significantly (*p* < 0.001) in non-sprayed eggs at 28 °C, while T4 (2–8 °C with spray) exhibited the least fluctuation, confirming the spray’s role in preserving yolk quality (Table 4). Higher temperatures accelerated pH changes, but the film-forming spray mitigated this effect. Sprayed eggs at 2–8 °C (T4) showed stable pH over time, enhancing long-term freshness (Table 4).

#### 3.4.2. Haugh Unit, Albumen Height and Weight%

Haugh unit values declined significantly (*p* < 0.001) in T1 (84.80 to 27.82), indicating severe degradation. Sprayed eggs at 2–8 °C (T4) showed a minimal decline (84.31 to 70.72), extending shelf life by three weeks compared to untreated eggs (Table 5). At 28 °C without spray (T1), albumen height significantly decreased (7.03 mm to 1.21 mm, *p* < 0.001). Sprayed eggs at 2–8 °C (T4) retained better height (6.95 mm to 4.85 mm), reinforcing the spray’s effectiveness (Table 5). The effect of the film-forming spray on albumen weight was studied in eggs stored at 28 °C and 2–8 °C for 42 days (Supplementary Table S4). In non-sprayed eggs at 28 °C (T1), albumen weight dropped from 52.6% to 42.5% (*p* < 0.001). This sharp decline shows the impact of high temperatures on moisture loss and albumen quality. In non-sprayed eggs at 2–8 °C (T2), albumen weight decreased from 52.6% to 49.78%. In sprayed eggs at 28 °C (T3), albumen weight dropped from 52.6% to 44.83%, showing a slower decline than T1. In sprayed eggs at 2–8 °C (T4), albumen weight slightly decreased from 52.6% to 51.6% (Supplementary Table S4).

#### 3.4.3. Yolk Weight (%), Index and Color

Due to albumen moisture loss, yolk weight increased significantly (*p* < 0.001) in non-sprayed eggs at 28 °C. Spray application (T3 and T4) mitigated this effect, stabilizing yolk weight (Table 5). T1 showed the highest decline (0.020), while T4 (0.382) maintained the yolk structure effectively, demonstrating the protective role of spray and refrigeration (Table 5). Yolk pigmentation faded significantly (*p* < 0.001) in T1 (8 to 4), whereas T4 (8 to 7) exhibited superior color retention, emphasizing the spray’s role in preventing oxidative degradation (Table 5).

### 3.5. Microbial Load

At 28 °C without spray (T1), microbial growth surged from 3.82 to 7.74 log cfu/mL by Day 42. In contrast, sprayed eggs at 2–8 °C (T4) exhibited a significantly lower TPC, reinforcing the film-forming spray’s antimicrobial efficacy in prolonging egg shelf life (Table 6). Similarly, we assessed the effects of the film-forming spray on *E. coli* counts in eggs stored at different temperatures and durations. Initial counts on Day 0 were low across all groups: T1 (28 °C) at 0.23 ± 0.04 cfu/mL, T2 (2–8 °C) at 0.26 ± 0.03 cfu/mL, T3 (28 °C + spray) at 0.24 ± 0.03 cfu/mL, and T4 (2–8 °C + spray) at 0.24 ± 0.04 cfu/mL. By Day 6, T1 increased significantly to 0.82 ± 0.03 cfu/mL, while T2 rose moderately to 0.43 ± 0.04 cfu/mL; T3 and T4 recorded 0.53 ± 0.04 cfu/mL and 0.40 ± 0.04 cfu/mL, respectively. Over time, T1 reached 4.50 ± 0.04 cfu/mL by Day 42, while T2 and T3 recorded 2.71 ± 0.05 cfu/mL and 3.38 ± 0.04 cfu/mL, suggestive of an inhibited microbial load at the lower temperatures (Table 6). T4, despite low-temperature storage and film-forming spray treatment, maintained a microbial count of 2.28 ± 0.05 cfu/mL. Statistical analysis revealed significant differences among treatment groups from Day 12 onward (*p* < 0.05), emphasizing the influence of storage temperature and phytobiotic application on *E. coli* proliferation in egg.

The film-forming spray in the concentration of 0.25 mg, 0.5 mg, 0.75 mg and 1.0 mg on testing with *Samlonella typhimurium* strain 2959 resulted in a zone diameter of 8 mm, 11 mm, 15 mm and 16 mm, respectively. Similarly, on testing with *Samlonella typhimurium* strain 22NSC003 in a similar concentration of the spray resulted zones of inhibition of 6 mm, 8 mm, 10 mm and 13 mm, respectively (Supplementary Figure S3).

## 4. Discussion

This study explores, develops and evaluates a film-forming spray used to extend the poultry industry’s egg shelf life and analyzes its antimicrobial effects. The formulations incorporated PVA, PEG, and phytochemicals for various test concentrations. PVA, widely used in high-barrier food packaging, acts as an antibacterial layer, preventing oxygen, moisture, and bacterial contamination [39]. Combined with guanidinium polymer, PVA enhances transparency, mechanical strength, and antibacterial efficacy, making it ideal for films and hydrogels [40]. Phytobiotic components like glucosides, polysaccharides, phenolic acids, and more are utilized as film-forming agents in edible films and coatings, enhancing their properties and bioactivity [41]. Phytochemicals derived from plants exhibit potential as film-forming agents due to their ability to inhibit biofilm formation. These natural bioactive compounds can interact with edible films and coatings, enhancing their properties and functionality [42]. In a study, Monton et al. (2021) optimized a Trisattakula herbal recipe, identifying dried *Nigella sativa* seeds as the most effective antibacterial component [43]. The best film-forming polymeric solution (FFPS) formulation, containing 9.2% *N. sativa* extract, demonstrated potent activity against *Staphylococcus aureus* and *S. epidermidis*. These findings suggest that the FFPS with *N. sativa* extract could be a promising alternative antibacterial treatment for skin infections caused by these pathogens [43].

Studies have shown that PEG and other plasticizers like glycerol and sorbitol can improve the characteristics of edible films, making them elastic, flexible, and less fragile [44]. PEG 400 was utilized as a plasticizer to prepare films using the mucilage of *Moringa oleifera*, resulting in films with satisfactory drying properties [45]. Various combinations of plasticizers and polymers are formulated based on their compatibility and film-forming properties. The key to preparing the film-forming spray is determining the ideal film thickness, which depends on polymer concentration. Mechanical and integrity tests are conducted to establish the threshold strength of the film. The synergistic effect between the PVA film and phytobiotics lies in their complementary functionalities [46,47]. PVA is a biocompatible, film-forming polymer that provides a stable matrix for the uniform encapsulation and sustained release of phytoconstituents. This matrix enhances the adhesion and coverage over the egg surface while simultaneously sealing pores to limit gas exchange and moisture loss [46,47]. Phytobiotics, rich in antimicrobial and antioxidant compounds, exert targeted action by disrupting bacterial membranes and inhibiting microbial growth. Together, the PVA film stabilizes and prolongs the bioactivity of the phytobiotics, leading to improved microbial inhibition and an extended shelf life of the eggs [46,47,48].

Six formulations (F1–F6) were synthesized (Supplementary Figure S1) and evaluated for the qualitative and physical appearance analysis. The evaporation time ranged from 30 s to 55 min, while the drying time varied from 1.31 to 3.37 min. pH levels were slightly acidic to neutral, ensuring compatibility. Film thickness (0.03–0.12 mm) influenced barrier efficacy, while all formulations exhibited a glossy, translucent appearance. Parra et al. (2024) developed a novel polymeric coating using electrolyzed acidic water (EAW), with hypochlorous acid as the active compound [49]. SEM analysis revealed that the coating had a thickness of 2.9 µm (0.0029 mm) on eggs from young and adult hens, while it measured 2.6 µm (0.0026 mm) on eggs from older hens [49]. Their findings demonstrated that the polymeric coating significantly extended the shelf life of eggs and exhibited antimicrobial properties, aligning with the results of the present study. Water solubility varied, with F1 and F2 being easily removable, F3 and F4 moderately so, and F5 and F6 more resistant. Film integrity ranged from intact (F1 and F2) to minor cracks (F3 and F4) and flaking (F5 and F6). Viscosity spanned from glycerol-like to syrup-like, affecting spreadability. Based on physical and qualitative attributes, F4 and F5 were selected for further characterization, including zeta size, potential, FTIR, and SEM analysis.

FTIR analysis confirms the presence of O-H/N-H and C=O functional groups in F5, with additional peaks at lower wave numbers indicating alkyl or phosphate compounds that may enhance betaine stability and efficacy in formulations [50,51]. Materials such as betaine could potentially be used as antimicrobial agents in a dose-dependent manner by coating surfaces [50]. SEM analysis reveals a uniform structure with smaller primary particles. The denser, more compact morphology of F5 enhances structural integrity, making it ideal for applications requiring consistency. The zeta size distribution analysis of formulations (F4 and F5) as “Good” in measurement reliability confirmed their uniformity. Additionally, the zeta potential analysis indicates stable dispersion properties attributed to a negative charge, further supporting their stability.

Simultaneously, the spray’s effectiveness over time is assessed under different storage conditions. According to data from the Indian Meteorological Department (IMD), India’s average annual ambient temperature typically ranges between 24 °C and 28 °C. However, in certain regions, particularly in commercial practices, eggs are stored under cold storage conditions, often maintained between 2 °C and 15 °C, to extend shelf life and maintain quality. Considering these contrasting storage environments, the present study was conducted under two representative temperature conditions: ambient (28 °C) and refrigerated (2 °C to 8 °C). The coating effectively prolonged shelf life, maintaining quality for up to 45 days under refrigeration and over 30 days at room temperature. Eggs stored at 28 °C without treatment (T1) experienced the highest weight loss, increased air cell diameter, rapid albumen and yolk quality deterioration, and substantial microbial growth, leading to severe spoilage by Day 42. Refrigeration (T2) effectively slowed quality degradation, while the film-forming spray at 28 °C (T3) provided moderate protection against environmental stressors [52]. The best extended shelf life was achieved with eggs treated with the spray and stored at 2–8 °C (T4), maintaining superior freshness, albumen stability, yolk integrity, and microbial safety (Supplementary Figure S2) [53]. This outcome highlights the synergistic effect of the film-forming spray when combined with refrigeration. Sensory evaluation further confirmed that T4 had the highest acceptability, reinforcing the effectiveness of this preservation strategy in extending shelf life and reducing post-harvest losses. This study demonstrates the significant antimicrobial efficacy of the film-forming spray in preserving egg quality under varying storage conditions. Eggs stored at 28 °C without treatment (T1) showed a marked increase in total plate count (TPC) and *E. coli* levels over 42 days, indicating rapid microbial proliferation under ambient conditions. In contrast, eggs treated with the spray and stored at 2–8 °C (T4) consistently exhibited the lowest microbial counts, underscoring the synergistic effect of phytobiotic coating and refrigeration in minimizing spoilage. From Day 12 onward, statistical analysis confirmed significant differences across treatment groups (*p* < 0.05), validating the role of storage temperature and spray application. The film’s direct antimicrobial action was further supported by in vitro assays, where increasing spray concentrations produced larger zones of inhibition against *Salmonella typhimurium* strains, reaching up to 16 mm at 1.0 mg concentration. The film-forming spray was not tested directly against *Salmonella spp.* in egg samples, as farmers and cold storage facilities maintain routine monthly *Salmonella* certification per Indian regulatory standards. However, the spray was evaluated against poultry strains of *Salmonella typhimurium,* resulting in a concentration-dependent inhibition of growth.

Oliveira et al. (2020) investigated the impact of a pectin biofilm on preserving eggs stored under refrigerated and room temperature conditions for five weeks [54]. The study assessed egg quality based on weight loss, albumen height, Haugh unit (HU), and yolk index (YI). The results showed that at room temperature, coated eggs had significantly higher HU values (71.27 ± 10.78) than uncoated eggs (59.11 ± 15.97). Similarly, the YI was slightly higher in coated eggs (0.37 ± 0.16) than in uncoated ones (0.35 ± 0.16). The pectin biofilm with refrigeration effectively preserved egg quality over the 35-day storage period [54].

The sensory quality and overall consumer acceptability of eggs were evaluated after storage under different temperature conditions. At the end of each storage week, eggs were boiled and presented to a panel of trained evaluators, who assessed key sensory attributes such as odor, color, and texture using a standardized hedonic scale. An overall acceptability score was then derived by collectively considering all individual parameters. Each attribute was rated according to the scoring scale provided in the table, with higher scores indicating greater acceptability. This evaluation was conducted every sixth day to monitor changes in sensory quality over time, reflecting the impact of different storage conditions (Supplementary Table S5).

This novel film-forming spray demonstrates strong potential in mitigating post-harvest losses by enhancing the shelf life and microbial safety of eggs. While the study focused on Indian conditions, this technology holds broad global relevance, particularly for countries facing similar challenges in egg preservation due to inadequate cold chain infrastructure. Many countries, particularly those with limited cold chain infrastructure or significant rural–urban supply gaps, face similar challenges in preserving egg quality during storage and transportation. Its ease of application, cost-effectiveness, and ability to maintain egg quality under both refrigerated and ambient conditions make it a scalable solution for small- to large-scale producers worldwide. Future research should explore its adoption in diverse climatic and logistical settings to validate its global utility [55].

## 5. Conclusions

The novel synthesized film-forming spray significantly enhances egg shelf life, reducing microbial load with sustainable approaches. At 28 °C, treated eggs remain viable for up to 30 days, while refrigeration (2–8 °C) extends storage beyond 42 days, potentially up to 60 days. The developed formulation offers significant benefits to the poultry industry by enhancing the storage stability of table eggs, reducing spoilage, and mitigating microbial contamination. By minimizing losses incurred during storage, this innovation leads to positive economic outcomes for farmers, wholesalers, and retailers. Ultimately, this solution enhances the overall efficiency and sustainability of the egg industry while meeting consumer demands for longer-lasting, superior-quality eggs. Future research should be directed to optimize formulation stability and evaluate long-term sensory and nutritional impacts for commercial applications. Additionally, exploring its efficacy on other perishable food items could further enhance food preservation requirements.

This film-forming spray could be applied to breeder eggs for future research to reduce contamination and spoilage effectively. Since breeder eggs are significantly more expensive than broiler eggs, extending their shelf life is especially valuable. Additionally, egg production and distribution are often uneven across different areas in India and other economically challenged regions. This spray could be crucial in minimizing spoilage during transportation across long distances. Moreover, eggs stored in cold conditions for extended periods, such as 60, 75, or 90 days, can benefit from applying this spray immediately after removal from storage. The film-forming spray helps to decrease contamination and spoilage, thereby preserving both the internal and external quality of eggs during prolonged storage. Overall, this spray presents a promising solution to enhance egg storage and transportation practices, reduce post-harvest losses, and improve consumer satisfaction. Future studies could explore optimizing its use across various egg types and storage durations to maximize its practical benefits.

## 6. Patents

The patent application no. 202311066448. The phytobiotics were procured from Elixir Extracts Pvt. Ltd., Kerala, India.

## Figures and Tables

**Table 1:** Evaluation of physical appearance parameters of film-forming spray. These attributes provide insight into each spray formulation’s aesthetic and functional qualities.

Formulation	Water Washability	Cosmetic Appearance	Integrity	Viscosity
F1	1	2	2	1
F2	1	2	2	1
F3	2	2	3	1
F4	2	2	3	2
F5	3	3	3	2
**Grading**	**1:** Easily washable	**1:** Shiny and transparent	**1:** Crack	**1:** Water-like
	**2:** Moderately washable	**2:** Shiny and translucent	**2:** Flaking	**2:** Glycerol-like
	**3:** Poorly washable	**3:** Dull and opaque	**3:** Intact	**3:** Syrup-like

**Table 2:** Qualitative assessment of film-forming spray.

Formulation	Evaporation Time	Drying Time	pH	Volume Per Spray	Thickness
	(Min: Sec)	(Min)		(mL)	(mm)
F1	30	1.31	5.78	0.1	0.03
F2	31	3.37	5.87	0.1	0.07
F3	34	2.54	5.57	0.1	0.07
F4	38	2.06	5.62	0.1	0.09
F5	55	2.13	5.65	0.1	0.11
F6	51	2.42	5.72	0.1	0.12

**Table 3:** Effect of film-forming spray on egg’s weight loss (%) under different storage periods and temperatures.

Duration/ Temperature	6th Day	12nd Day	18th Day	24th Day	30th Day	36th Day	42nd Day	*p* Value
T1 (28 °C)	2.81 ± 0.62 ^A^	5.69 ± 0.64 ^bB^	7.4 ± 0.62 ^B^	9.58 ± 0.73 ^cC^	12.25 ± 0.73 ^cC^	14.73 ± 0.83 ^cD^	15.58 ± 0.82 ^cE^	0.000
T2 (2–8 °C)	1.66 ± 0.81 ^A^	2.25 ± 0.27 ^bAB^	3.29 ± 0.7 ^AB^	3.79 ± 0.29 ^abB^	5.02 ± 0.77 ^abB^	6.06 ± 0.48 ^bBC^	6.99 ± 0.48 ^bBC^	0.000
T3 (28 °C) + Spray	1.09 ± 0.72 ^A^	2.4 ± 0.42 ^bAB^	3.63 ± 0.36 ^AB^	4.86 ± 0.4 ^bAB^	7.64 ± 2.76 ^bcB^	8.56 ±0.52 ^bBC^	8.87 ± 0.52 ^bC^	0.000
T4 (2–8 °C) + Spray	0.8 ± 0.52 ^A^	1.33 ± 0.25 ^aAB^	1.36 ± 0.36 ^aAB^	2.01 ± 0.29 ^AB^	2.29 ± 0.33 ^aB^	3.55 ± 0.34 ^aB^	4.52 ± 0.33 ^aBC^	0.000
*p* Value	0.164	0.000	0.000	0.000	0.000	0.000	0.000	

N = 10; mean ± SE; the mean values between columns with different superscripts a, b, c, are significantly different (*p* < 0.001); the mean values between rows with different superscripts A, B, C, D, E, are significantly different (*p* < 0.001).

**Table 4:** Effect of film-forming spray on air cell diameter and specific gravity, pH of albumen, yolk, and whole egg under different storage periods and temperatures.

Air Cell Diameter									
Duration/ Temperature	0th Day	6th Day	12nd Day	18th Day	24th Day	30th Day	36th Day	42nd Day	*p* Value
T1 (28 °C)	16.48 ± 0.21 ^A^	23.47 ± 0.24 ^cB^	26.88 ± 0.23 ^dB^	28.97 ± 0.29 ^dBC^	30.35 ± 0.29 ^cC^	31.89 ± 0.27 ^cCD^	33.02 ± 0.31 ^cD^	33.68 ± 0.32 ^cDE^	0.000
T2 (2–8 °C)	17.86 ± 0.15 ^A^	20.45 ± 0.14 ^bB^	22.27 ± 0.18 ^bB^	24.44 ± 0.2 ^bBC^	25.57 ± 0.19 ^bBC^	26.24 ± 0.17 ^bC^	27.87 ± 0.25 ^bC^	28.63 ± 0.33 ^bCD^	0.000
T3 (28 °C) + Spray	17.48 ± 0.18 ^A^	20.64 ± 0.26 ^bB^	23.51 ± 0.3 ^cB^	25.29 ± 0.32 ^cB^	27.04 ± 0.33 ^bBC^	28.59 ± 0.35 ^bC^	29.27 ± 0.37 ^bCD^	29.85 ± 0.38 ^bCD^	0.000
T4 (2–8 °C) + Spray	16.73 ± 0.22 ^A^	19.12 ± 0.2 ^aB^	21.07 ± 0.33 ^aB^	21.9 ± 0.34 ^aB^	23.15 ± 0.26 ^aB^	24.54 ± 0.33 ^aBC^	24.94 ± 0.32 ^aBC^	25.44 ± 0.32 ^aC^	0.023
*p* Value	0.245	0.000	0.000	0.000	0.000	0.000	0.000	0.000	
**Specific gravity**									
T1 (28 °C)	1.092 ± 0.001 ^A^	1.067 ± 0.001 ^bcB^	1.055 ± 0.001 ^bcC^	1.048 ± 0.01 ^bD^	1.045 ± 0.003 ^cE^	1.044 ± 0.00 ^cF^	1.043 ± 0.00 ^cG^	1.04 ± 0.00 ^cH^	0.000
T2 (2–8 °C)	1.094 ± 0.002 ^A^	1.081 ± 0.001 ^aAB^	1.072 ± 0.001 ^abB^	1.066 ± 0.002 ^abBC^	1.061 ± 0.001 ^abC^	1.06 ± 0.001 ^abC^	1.059 ± 0.001 ^abC^	1.056 ± 0.001 ^abCD^	0.000
T3 (28 °C) + Spray	1.094 ± 0.001 ^A^	1.075 ± 0.001 ^bB^	1.066 ± 0.002 ^bC^	1.061 ± 0.001 ^abCD^	1.056 ± 0.001 ^bDE^	1.055 ± 0.001 ^bDE^	1.054 ± 0.002 ^bDE^	1.051 ± 0.001 ^bDE^	0.000
T4 (2–8 °C) + Spray	1.094 ± 0.001 ^A^	1.091 ± 0.003 ^aAB^	1.082 ± 0.001 ^aB^	1.076 ± 0.001 ^aBC^	1.071 ± 0.001 ^aBC^	1.07 ± 0.002 ^aBC^	1.069 ± 0.001 ^aC^	1.066 ± 0.001 ^aC^	0.000
*p* Value	0.245	0.003	0.006	0.001	0.002	0.000	0.000	0.000	
**Albumen pH**									
T1 (28 °C)	7.86 ± 0.16 ^A^	8.01 ± 0.17 ^B^	8.18 ± 0.17 ^C^	8.46 ± 0.17 ^D^	8.78 ± 0.18 ^Ea^	9.12 ± 0.19 ^Fa^	9.46 ± 0.20 ^Ga^	9.82 ± 0.20 ^Ha^	0.015
T2 (2–8 °C)	7.87 ± 0.16 ^A^	7.89 ± 0.16 ^A^	7.93 ± 0.16 ^A^	7.99 ± 0.17 ^A^	8.04 ± 0.17 ^ABb^	8.09 ± 0.17 ^Abb^	8.13 ± 0.17 ^Bc^	8.20 ± 0.17 ^Cc^	0.039
T3 (28 °C) + Spray	7.88 ± 0.16 ^A^	7.97 ± 0.16 ^AB^	8.07 ± 0.17 ^BC^	8.17 ± 0.17 ^CD^	8.26 ± 0.17 ^Dab^	8.36 ± 0.17 ^Eab^	8.46 ± 0.18 ^Eb^	8.53 ± 0.18 ^Fb^	0.014
T4 (2–8 °C) + Spray	7.88 ± 0.16 ^A^	7.91 ± 0.16 ^A^	7.92 ± 0.16 ^A^	7.94 ± 0.16 ^A^	7.95 ± 0.16 ^ABb^	7.97 ± 0.16 ^ABc^	7.98 ± 0.16 ^Bcd^	8.00 ± 0.16 ^Bd^	0.048
*p* Value	0.998	0.948	0.659	0.138	0.007	0.06	0.000	0.000	
**Yolk pH**									
T1 (28 °C)	6.41 ± 0.07 ^A^	6.54 ± 0.08 ^B^	6.67 ± 0.08 ^C^	6.80 ± 0.08 ^D^	6.94 ± 0.08 ^E^	7.07 ± 0.08 ^Fa^	7.20 ± 0.08 ^Ga^	7.34 ± 0.09 ^Ha^	0.000
T2 (2–8 °C)	6.44 ± 0.13 ^A^	6.46 ± 0.13 ^A^	6.49 ± 0.13 ^A^	6.54 ± 0.13 ^AB^	6.58 ± 0.14 ^AB^	6.62 ± 0.14 ^ABb^	6.66 ± 0.14 ^ABb^	6.71 ± 0.14 ^Bc^	0.023
T3 (28 °C) + Spray	6.48 ± 0.06 ^A^	6.55 ± 0.06 ^AB^	6.61 ± 0.06 ^BC^	6.65 ± 0.06 ^CD^	6.70 ± 0.06 ^D^	6.74 ± 0.06 ^Dab^	6.77 ± 0.06 ^DEab^	6.82 ± 0.06 ^Eb^	0.011
T4 (2–8 °C) + Spray	6.46 ± 0.04 ^A^	6.47 ± 0.04 ^A^	6.49 ± 0.04 ^A^	6.51 ± 0.04 ^A^	6.53 ± 0.04 ^AB^	6.55 ± 0.05 ^ABb^	6.57 ± 0.05 ^ABc^	6.59 ± 0.05 ^Bc^	0.080
*p* Value	0.946	0.855	0.855	0.388	0.099	0.012	0.001	0.000	
**Whole Egg pH**									
T1 (28 °C)	7.07 ± 0.04 ^A^	7.21 ± 0.04 ^B^	7.35 ± 0.04 ^Ca^	7.50 ± 0.04 ^Da^	7.65 ± 0.04 ^Ea^	7.79 ± 0.05 ^Fa^	7.94 ± 0.05 ^Ga^	8.09 ± 0.05 ^Ha^	0.000
T2 (2–8 °C)	7.09 ± 0.04 ^A^	7.11 ± 0.04 ^A^	7.15 ± 0.04 ^Abb^	7.20 ± 0.04 ^ABa^	7.24 ± 0.04 ^ABb^	7.29 ± 0.04 ^Bb^	7.32 ± 0.04 ^ab^	7.38 ± 0.04 ^Cb^	0.037
T3 (28 °C) + Spray	7.08 ± 0.03 ^A^	7.16 ± 0.03 ^B^	7.23 ± 0.03 ^BCa^	7.27 ± 0.03 ^CDab^	7.32 ± 0.03 ^Dbc^	7.37 ± 0.03 ^Dc^	7.40 ± 0.03 ^DEb^	7.46 ± 0.03 ^Eb^	0.014
T4 (2–8 °C) + Spray	7.08 ± 0.05 ^A^	7.10 ± 0.05 ^A^	7.12 ± 0.05 ^Aab^	7.14 ± 0.05 ^Ab^	7.16 ± 0.05 ^ABc^	7.18 ± 0.05 ^Bd^	7.21 ± 0.05 ^BCc^	7.23 ± 0.05 ^Cc^	0.056
*p* Value	0.982	0.157	0.002	0.005	0.000	0.000	0.000	0.000	

N = 10; mean ± SE; the mean values between columns with different superscripts a, b, c, d, are significantly different (*p* < 0.001); the mean values between rows with different superscripts A, B, C, D, E, F, G and H are significantly different (*p* < 0.001).

**Table 5:** Effect of film-forming spray on Haugh unit and albumen height, yolk weight (%), index, and color under different storage periods and temperatures.

Haugh Unit									
Duration/ Temperature	0th Day	6th Day	12nd Day	18th Day	24th Day	30th Day	36th Day	42nd Day	*p* Value
T1 (28 °C)	84.80 ± 0.76 ^A^	77.79 ± 1.43 ^bB^	70.72 ± 1.08 ^C^	59.74 ± 2.77 ^cD^	51.01 ± 3.16 ^cDE^	46.21 ± 3.16 ^cE^	33.26 ± 1.27 ^cF^	27.82 ± 1.17 ^dG^	0.000
T2 (2–8 °C)	84.03 ± 0.81 ^A^	80.55 ± 1.04 ^aAB^	77.79 ± 1.54 ^aB^	73.08 ± 1.53 ^bB^	71.36 ± 1.84 ^bBC^	67.27 ± 2.08 ^bC^	64.81 ± 2.92 ^bC^	65.21 ± 2.19 ^bD^	0.014
T3 (28 °C) + Spray	84.92 ± 0.81 ^A^	83.46 ± 0.86 ^aAB^	75.66 ± 0.76 ^aB^	71.14 ± 1.85 ^bCD^	66.80 ± 2.64 ^bD^	62.69 ± 4.71 ^bDE^	58.81 ± 3 ^bE^	49.19 ± 3.14 ^cEF^	0.000
T4 (2–8 °C) + Spray	84.31 ± 1.25 ^A^	83.46 ± 1.22 ^aA^	80.28 ± 1.91 ^aB^	78.34 ± 1.29 ^aB^	77.30 ± 1.6 ^a^	75.90 ± 2.59 ^a^	74.11 ± 1.98 ^a^	70.72 ± 1.6 ^a^	0.002
*p* Value	0.955	0.000	0.000	0.000	0.000	0.000	0.000	0.000	
**Albumen height (mm)**									
T1 (28 °C)	7.03 ± 0.16 ^A^	5.76 ± 0.13 ^cB^	4.72 ± 0.10 ^cC^	3.59 ± 0.08 ^dD^	2.73 ± 0.06 ^dE^	2.07 ± 0.05 ^dF^	1.58 ± 0.03 ^dG^	1.21 ± 0.03 ^dH^	0.000
T2 (2–8 °C)	6.90 ± 0.15 ^A^	6.35 ± 0.14 ^aAB^	5.71 ± 0.13 ^bAB^	5.14 ± 0.11 ^bB^	4.73 ± 0.10 ^bBC^	4.35 ± 0.1 ^bC^	4.01 ± 0.09 ^bCD^	3.88 ± 0.09 ^bD^	0.018
T3 (28 °C) + Spray	6.96 ± 0.12 ^A^	6.13 ± 0.10 ^abB^	5.39 ± 0.09 ^abAB^	4.75 ± 0.08 ^cB^	4.18 ± 0.07 ^cC^	3.67 ± 0.06 ^cD^	3.23 ± 0.05 ^cD^	2.85 ± 0.05 ^cDE^	0.000
T4 (2–8 °C) + Spray	6.95 ± 0.13 ^A^	6.60 ± 0.12 ^aAB^	6.27 ± 0.11 ^aB^	5.96 ± 0.11 ^aBC^	5.66 ± 0.11 ^aBC^	5.38 ± 0.12 ^aC^	5.11 ± 0.09 ^aCD^	4.85 ± 0.09 ^aD^	0.048
*p* Value	0.936	0.003	0.005	0.000	0.000	0.000	0.000	0.000	
**Yolk Weight (%)**									
T1 (28 °C)	29.65 ± 1.11 ^A^	28.47 ± 1.16 ^AB^	29.33 ± 1.2 ^AB^	30.21 ± 1.23 ^B^	31.11 ± 1.27 ^B^	32.05 ± 1.31 ^C^	33.01 ± 1.35 ^D^	34 ± 1.39 ^aE^	0.000
T2 (2–8 °C)	28.92 ± 0.79 ^A^	28.22 ± 1.04 ^A^	28.57 ± 1.05 ^A^	28.92 ± 1.07 ^A^	29.29 ± 1.08 ^B^	29.65 ± 1.09 ^B^	29.73 ± 1.1 ^BC^	29.8 ± 1.1 ^bC^	0.024
T3 (28 °C) + Spray	28.78 ± 0.68 ^A^	28.92 ± 0.97 ^A^	29.28 ± 0.99 ^A^	29.65 ± 1 ^AB^	30.02 ± 1.01 ^B^	30.39 ± 1.02 ^BC^	30.47 ± 1.03 ^C^	30.55 ± 1.03 ^abD^	0.004
T4 (2–8 °C) + Spray	28.9 ± 0.72	28.92 ± 0.73	28.95 ± 0.73	28.97 ± 0.73	29 ± 0.73	29.03 ± 0.73	29.05 ± 0.73	29.08 ± 0.73 ^c^	0.329
*p* Value	0.886	0.942	0.945	0.789	0.450	0.228	0.068	0.024	
**Yolk Index**									
T1 (28 °C)	0.444 ± 0.007 ^A^	0.398 ± 0.005 ^cB^	0.24 ± 0.006 ^cC^	0.18 ± 0.005 ^dD^	0.14 ± 0.009 ^dE^	0.091 ± 0.01 ^dF^	0.044 ± 0.007 ^dG^	0.020 ± 0.002 ^dH^	0.000
T2 (2–8 °C)	0.44 ± 0.008 ^A^	0.427 ± 0.007 ^aA^	0.406 ± 0.007 ^aAB^	0.403 ± 0.011 ^aAB^	0.393 ± 0.006 ^aAB^	0.384 ± 0.009 ^aB^	0.356 ± 0.015 ^aC^	0.363 ± 0.01 ^aC^	0.032
T3 (28 °C) + Spray	0.444 ± 0.007 ^A^	0.391 ± 0.007 ^bcB^	0.373 ± 0.006 ^bBC^	0.371 ± 0.01 ^bBC^	0.361 ± 0.006 ^bC^	0.353 ± 0.008 ^bC^	0.327 ± 0.014 ^bD^	0.334 ± 0.009 ^bD^	0.000
T4 (2–8 °C) + Spray	0.444 ± 0.007 ^A^	0.428 ± 0.007 ^aA^	0.408 ± 0.007 ^aA^	0.406 ± 0.011 ^aA^	0.396 ± 0.006 ^aAB^	0.387 ± 0.009 ^aAB^	0.387 ± 0.009 ^aAB^	0.382 ± 0.007 ^aB^	0.021
*p* Value	0.966	0.000	0.000	0.000	0.000	0.000	0.000	0.000	
**Yolk color**									
T1 (28 °C)	8 ± 0.18 ^A^	8 ± 0.17 ^A^	7 ± 0.36 ^Bb^	6 ± 0.24 ^Cc^	6 ± 0.26 ^Cc^	5 ± 0.32 ^Dc^	5 ± 0.28 ^Dd^	4 ± 0.22 ^Ed^	0.000
T2 (2–8 °C)	8 ± 0.17 ^A^	8 ± 0.17 ^A^	8 ± 0.24 ^Aa^	8 ± 0.22 ^Aa^	8 ± 0.24 ^Aa^	8 ± 0.22 ^Aa^	7 ± 0.26 ^Bb^	6 ± 0.28 ^Bb^	0.057
T3 (28 °C) + Spray	8 ± 0.16 ^A^	7 ± 0.20 ^B^	8 ± 0.14 ^Aa^	7 ± 0.28 ^Bb^	7 ± 0.22 ^Bb^	6 ± 0.22 ^Cb^	6 ± 0.23 ^Cc^	5 ± 0.09 ^Dc^	0.000
T4 (2–8 °C) + Spray	8 ± 0.16 ^A^	8 ± 0.20 ^A^	8 ± 0.28 ^Ba^	8 ± 0.22 ^Aa^	8 ± 0.16 ^Aa^	8 ± 0.41 ^Ba^	8 ± 0.22 ^Aa^	7 ± 0.13 ^Ba^	0.098
*p* Value	0.973	0.041	0.002	0.000	0.000	0.000	0.000	0.000	

N = 10; mean ± SE; the mean values between columns with different superscripts a, b, c, d, are significantly different (*p* < 0.001); the mean values between rows with different superscripts A, B, C, D, E, F, G and H are significantly different (*p* < 0.001).

**Table 6:** Effect of film-forming spray on TPC (10^4^ cfu/mL) and *E. coli* count (10^4^ cfu/mL) under different storage periods and temperatures.

TPC (10^4^ cfu/mL)									
Duration/ Temperature	0th Day	6th Day	12nd Day	18th Day	24th Day	30th Day	36th Day	42nd Day	*p* Value
T1 (28 °C)	3.82 ± 0.15 ^A^	4.42 ± 0.12 ^B^	4.83 ± 0.13 ^bBC^	5.43 ± 0.14 ^cC^	5.93 ± 0.16 ^cD^	6.33 ± 0.14 ^cE^	6.94 ± 0.18 ^cEF^	7.74 ± 0.15 ^dF^	0.000
T2 (2–8 °C)	3.82 ± 0.10 ^A^	3.92 ± 0.08 ^A^	4.02 ± 0.11 ^aAB^	4.12 ± 0.09 ^abB^	4.22 ± 0.07 ^abB^	4.32 ± 0.08 ^abB^	4.52 ± 0.10 ^aBC^	4.73 ± 0.12 ^bC^	0.044
T3 (28 °C) + Spray	3.82 ± 0.20 ^A^	4.22 ± 0.18 ^AB^	4.52 ± 0.17 ^bB^	4.93 ± 0.19 ^bBC^	5.43 ± 0.15 ^bC^	5.83 ± 0.20 ^bCD^	6.33 ± 0.21 ^bD^	6.74 ± 0.18 ^cE^	0.000
T4 (2–8 °C) + Spray	3.82 ± 0.05	3.82 ± 0.07	3.92 ± 0.09 ^a^	4.02 ± 0.10 ^a^	4.12 ± 0.11 ^a^	4.19 ± 0.12 ^a^	4.22 ± 0.09 ^a^	4.32 ± 0.11 ^a^	0.067
*p* Value	0.997	0.051	0.036	0.000	0.000	0.000	0.000	0.000	
***E. coli* count (10^4^ cfu/mL)**									
T1 (28 °C)	0.23 ± 0.04 ^A^	0.82 ± 0.03 ^B^	1.68 ± 0.05 ^Cc^	2.36 ± 0.04 ^Dc^	2.8 ± 0.06 ^DEd^	3.79 ± 0.05 ^Ed^	4.25 ± 0.04 ^Fd^	4.50 ± 0.04 ^G^	0.000
T2 (2–8 °C)	0.26 ± 0.03 ^A^	0.43 ± 0.04 ^A^	1.02 ± 0.03 ^ABa^	1.52 ± 0.05 ^ABab^	1.81 ± 0.04 ^Bb^	2.28 ± 0.05 ^Bb^	2.53 ± 0.03 ^BCb^	2.71 ± 0.05 ^Cb^	0.041
T3 (28 °C) + Spray	0.24 ± 0.03 ^A^	0.53 ± 0.04 ^AB^	1.39 ± 0.05 ^B^	1.65 ± 0.04 ^Bb^	1.97 ± 0.05 ^BCbc^	2.62 ± 0.04 ^Cc^	2.98 ± 0.05 ^CDc^	3.38 ± 0.04 ^Dc^	0.035
T4 (2–8 °C) + Spray	0.24 ± 0.04	0.40 ± 0.04	0.97 ± 0.03 ^ab^	1.26 ± 0.05 ^a^	1.48 ± 0.04 ^a^	1.95 ± 0.03 ^a^	2.13 ± 0.04 ^a^	2.28 ± 0.05 ^a^	0.059
*p* Value	0.957	0.087	0.048	0.040	0.027	0.018	0.000	0.000	

N = 10; mean ± SE; the mean values between columns with different superscripts a, b, c, d, are significantly different (*p* < 0.001); mean values between rows with different superscripts A, B, C, D, E, F, and G are significantly different (*p* < 0.001).

## Data Availability

The original contributions presented in this study are included in the article/Supplementary Material. Further inquiries can be directed to the corresponding authors.

## Supplementary Materials

The following supporting information can be downloaded at: https://www.mdpi.com/article/10.3390/polym17152142/s1, Figure S1: Procedure for film-forming spray formulation for eggs; Figure S2: Application of Phytobiotic spray on eggs; Figure S3: In vitro antimicrobial effect of phytobiotic spray on two clinical isolates of *Salmonella typhimurium* (2959 and 22NSC003) of poultry origin, demonstrating concentration-dependent zone of inhibition on a nutrient agar plate; Table S1: Experimental design of film-forming sprayed eggs stored under different temperatures and duration periods; Table S2: Effect of film-forming spray on shape index under different storage periods and temperature; Table S3: Effect of film-forming spray on eggshell thickness (mm) under different storage periods and temperatures; Table S4: Effect of film-forming spray on albumen weight % under different storage periods and temperatures; Table S5: Based on these grading, the sensory quality and overall consumer acceptability of eggs were evaluated after storage under different temperature conditions.
