# Supplementary material for: Novel Film-Forming Spray: Advancing Shelf Life Extension and Post-Harvest Loss Reduction in Eggs
Source: Polymers (Basel). 2025 Aug 5;17(15):2142. doi: 10.3390/polym17152142 (PMC12349253; doi:10.3390/polym17152142)
Supplement: Supplementary file 1 [file polymers-17-02142-s001.zip › polymers-3688702-supplementary.pdf]

Supplementary Materials

Novel Film-Forming Spray: Advancing Shelf Life Extension and Post-Harvest Loss Reduction in Eggs

Nagesh Sonale [1], Rokade J. Jaydip [1,*], Akhilesh Kumar [2,*], Monika Madheswaran [3], Rohit Kumar [2], Prasad Wadajkar [1] and Ashok Kumar Tiwari [1]

[1] ICAR—Central Avian Research Institute, Izatnagar, Bareilly 243122, Uttar Pradesh, India; dr.nssonale@gmail.com (N.S.); prasadwadajkar@gmail.com (P.W.); ashok.tiwari1@icar.gov.in(A.K.T.)
[2] Division of Medicine, ICAR—Indian Veterinary Research Institute, Izatnagar, Bareilly 243122, Uttar Pradesh, India; rajputrohit883@gmail.com
[3] ICAR—Indian Agriculture Research Institute, Hazaribag 825405, Jharkhand, India; monika.m@icar.gov.in
* Correspondence: jaydeepvet@gmail.com (R.J.J.); akhilesh.kumar@icar.gov.in (A.K.)

Table S1. Experimental design of film-forming sprayed eggs stored under different temperatures and duration periods.

S.no	Treatment	Eggs/Replicate	No. of Eggs/Groups
T_1	Eggs kept at 28 °C (Control)	40/4	160
T_2	Eggs kept at (2–8 °C)	40/4	160
T_3	Eggs kept at 28 °C + Spray	40/4	160
T_4	Eggs kept at (2–8 °C) + Spray	40/4	160
	**Total Eggs**		**640**

Table S2. Effect of film-forming spray on shape index under different storage periods and temperature.

Storage Temp.	0th Day	6th Day	12th Day	18th Day	24th Day	30th Day	36th Day	42nd Day	*p* Value
T1 (28 °C)	76.58 ± 0.42	76.57 ± 0.38	76.33 ± 0.29	75.94 ± 0.47	75.92 ± 0.39	75.76 ± 0.30	75.63 ± 0.28	75.50 ± 0.33	0.652
T2 (2–8 °C)	76.50 ± 0.35	76.47 ± 0.32	76.23 ± 0.43	75.87 ± 0.35	75.70 ± 0.44	75.60 ± 0.40	75.50 ± 0.31	76.40 ± 0.29	0.572
T3 (28 °C + Spray)	76.55 ± 0.29	76.30 ± 0.41	76.43 ± 0.37	75.26 ± 0.42	75.44 ± 0.31	75.30 ± 0.35	75.15 ± 0.36	75.00 ± 0.40	0.538
T4 (2–8 °C + Spray)	76.40 ± 0.31	76.37 ± 0.37	76.63 ± 0.28	75.79 ± 0.30	75.16 ± 0.27	75.05 ± 0.29	75.95 ± 0.30	76.80 ± 0.32	0.652
p Value	0.601	0.893	0.575	0.94	0.64	0.58	0.53	0.49	

N=10; mean± SE the mean values between columns with different superscripts a,b,c,d,e were significantly different (p<0.001) mean values between rows with different superscripts A, B, C, D, E, F, G. were significantly different (p <0.001).

Table S3. Effect of film-forming spray on eggshell thickness (mm) under different storage periods and temperatures.

Storage Temp.	0th Day	6th Day	12th Day	18th Day	24th Day	30th Day	36th Day	42nd Day	*p* Value
T1 (28 °C)	0.35 ± 0.008	0.35 ± 0.005	0.35 ± 0.005	0.34 ± 0.005	0.34 ± 0.004	0.34 ± 0.006	0.33 ± 0.003	0.33 ± 0.007	0.898
T2 (2–8 °C)	0.35 ± 0.003	0.36 ± 0.004	0.36 ± 0.006	0.35 ± 0.006	0.35 ± 0.006	0.35 ± 0.005	0.34 ± 0.003	0.34 ± 0.005	0.624
T3(28 °C) + Spray	0.35 ± 0.005	0.35 ± 0.006	0.35 ± 0.006	0.35 ± 0.005	0.34 ± 0.004	0.34 ± 0.005	0.34 ± 0.003	0.33 ± 0.004	0.521
T4(2–8 °C) + Spray	0.35 ± 0.007	0.36 ± 0.007	0.36 ± 0.004	0.35 ± 0.005	0.35 ± 0.002	0.35 ± 0.005	0.34 ± 0.006	0.34 ± 0.008	0.698
p Value	0.898	0.624	0.521	0.698	0.753	0.432	0.987	0.215	

N=10; mean± SE the mean values between columns with different superscripts a,b,c,d,e were significantly different (p<0.001) mean values between rows with different superscripts A, B, C, D, E, F, G.. were significantly different (p <0.001).

Table S4. Effect of film-forming spray on albumen weight % under different storage periods and temperatures.

Storage Temp.	0th Day	6th Day	12th Day	18th Day	24th Day	30th Day	36th Day	42nd Day	*p* Value
T1 (28 °C)	52.6 ± 2.38^{A}	51.02 ± 2.31^{A}	49.49 ± 2.24^{A}	48.01 ± 2.17^{A}	46.57 ± 2.11^{A}	45.17 ± 2.04^{Ac}	43.81 ± 1.98^{Ac}	42.5 ± 1.92^{Ac}	0.000
T2 (2–8 °C)	52.6 ± 2.15^{A}	52.18 ± 2.13^{A}	51.82 ± 2.12^{A}	51.41 ± 2.10^{A}	51.00 ± 2.08^{A}	50.59 ± 2.07^{Aa}	50.18 ± 2.05^{Aab}	49.78 ± 2.04^{Aab}	0.022
T3(28 °C) + Spray	52.6 ± 1.96^{A}	51.41 ± 1.92^{A}	50.25 ± 1.88^{A}	49.12 ± 1.83^{A}	48.01 ± 1.79^{A}	46.92 ± 1.75^{Ab}	45.86 ± 1.71^{Ab}	44.83 ± 1.67^{Ab}	0.005
T4(2–8 °C) + Spray	52.6 ± 2.15^{A}	52.32 ± 2.14^{A}	52.2 ± 2.14^{A}	52.08 ± 2.13^{A}	51.96 ± 2.13^{A}	51.84 ± 2.12^{Aa}	51.72 ± 2.12^{Aa}	51.6 ± 2.11^{Aa}	0.085
p Value	0.969	0.777	0.479	0.227	0.085	0.026	0.007	0.005	

N=10; mean± SE the mean values between columns with different superscripts a,b,c,d,e were significantly different ($p<0.001$) mean values between rows with different superscripts A, B, C, D, E, F, G.. were significantly different ($p <0.001$).

Table S5. Based on these grading, the sensory quality and overall consumer acceptability of eggs were evaluated after storage under different temperature conditions.

Grade Point	Description
**8**	Excellent—Very highly acceptable
**7**	Very good—Highly acceptable
**6**	Good—Acceptable
5	Fair—Slightly acceptable
4	Just fair—Barely acceptable
3	Poor—Slightly unacceptable
2	Very poor—Unacceptable
1	Extremely poor—Highly unacceptable

Temperature /Duration	0th Day	6th Day	12th Day	18th Day	24th Day	*p* Value
T_1(Amb Temp)	8.00 ± 0.02^{A}	7.55 ± 0.05^{B}	6.50 ± 0.04^{BC}	5.53 ± 0.03^{cC}	4.70 ± 0.02^{cCD}	0.000
T_2 (4 °C)	8.00 ± 0.03^{A}	7.67 ± 0.04^{AB}	7.53 ± 0.01^{aAB}	7.49 ± 0.05^{abAB}	7.46 ± 0.04^{abAB}	0.0260
T_3(Amb Temp + Spray)	8.00 ± 0.02^{A}	7.72 ± 0.03^{AB}	6.95 ± 0.03^{B}	6.25 ± 0.02^{bBC}	5.63 ± 0.03^{bC}	0.000
T_4 (4 °C+ Spray)	8.00 ± 0.03	8.00 ± 0.04	8.00 ± 0.02	7.96 ± 0.03^{a}	7.92 ± 0.03^{a}	0.0480
***p* Value**	0.957	0.680	0.425	0.041	0.038	

N=,10 mean± SE the mean values between column with different superscript a,b,c,dare significantly different ($p < 0.05$) mean values between rows with different superscript A,B,C,D, are significantly different ($p < 0.05$).

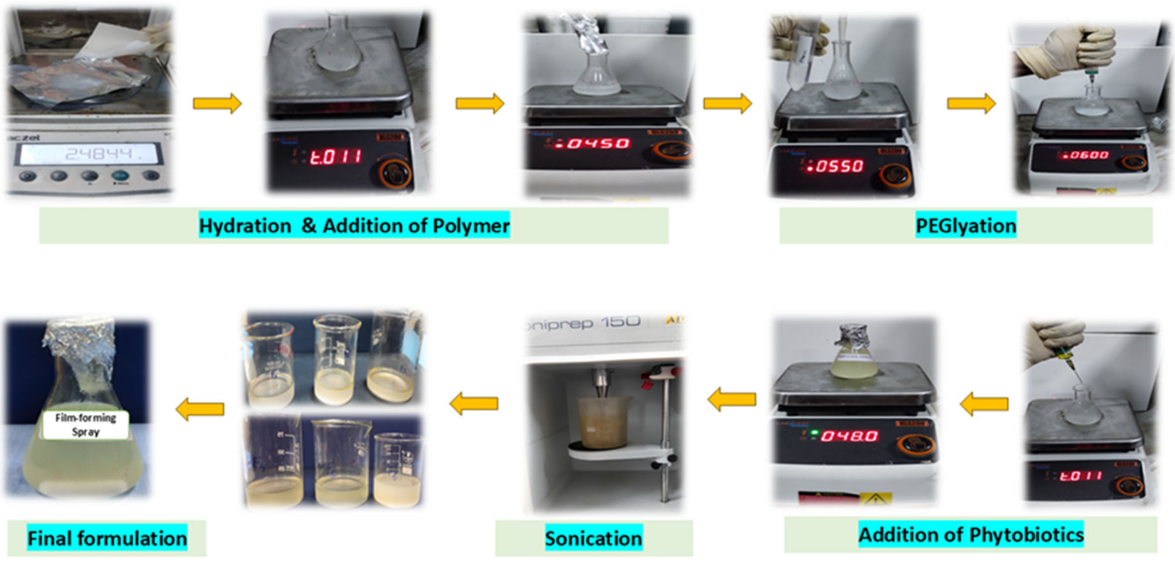

Figure S1. Procedure for film-forming spray formulation for eggs.

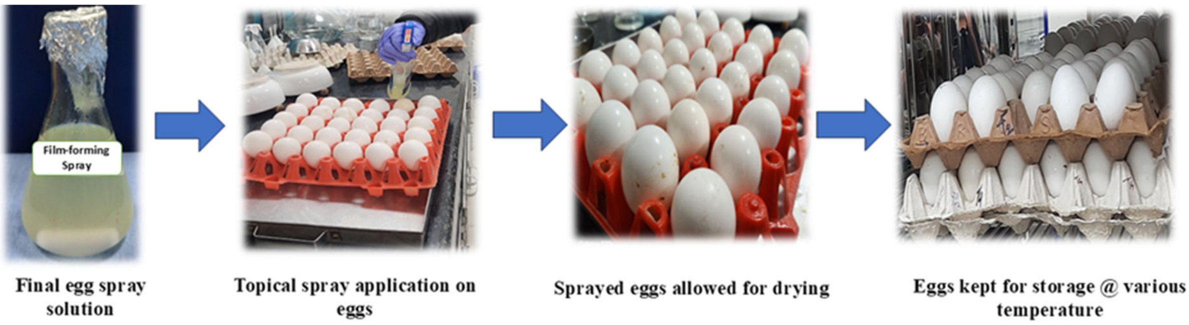

Figure S2. Application of Phytobiotic spray on eggs.

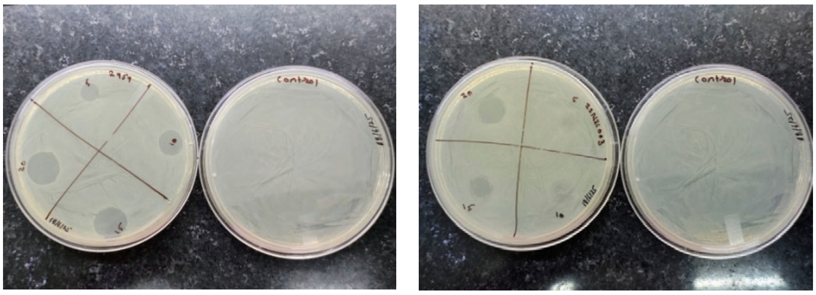

Figure S3. In vitro antimicrobial effect of phytobiotic spray on two clinical isolates of *Salmonella typhimurium* (2959 and 22NSC003) of poultry origin, demonstrating concentration-dependent zone of inhibition on a nutrient agar plate.
